# Printed Circuit Board Defect Detection Using Deep Learning via A Skip-Connected Convolutional Autoencoder

**DOI:** 10.3390/s21154968

**Published:** 2021-07-21

**Authors:** Jungsuk Kim, Jungbeom Ko, Hojong Choi, Hyunchul Kim

**Affiliations:** 1 Department of Biomedical Engineering, Gachon University, 191 Hambakmoe-ro, Incheon 2199, Korea; jungsuk@gachon.ac.kr (J.K.); rhwndqja@gachon.ac.kr (J.K.); 2 Department of Medial IT Convergence Engineering, Kumoh National Institute of Technology, 350-27 Gum-daero, Gumi 39253, Korea; 3 School of Information, University of California, Berkeley, 102 South Hall 4600, Berkeley, CA 94704, USA

**Keywords:** deep learning, autoencoder, detect detection, PCB defeat detection, printed circuit board manufacturing

## Abstract

As technology evolves, more components are integrated into printed circuit boards (PCBs) and the PCB layout increases. Because small defects on signal trace can cause significant damage to the system, PCB surface inspection is one of the most important quality control processes. Owing to the limitations of manual inspection, significant efforts have been made to automate the inspection by utilizing high resolution CCD or CMOS sensors. Despite the advanced sensor technology, setting the pass/fail criteria based on small failure samples has always been challenging in traditional machine vision approaches. To overcome these problems, we propose an advanced PCB inspection system based on a skip-connected convolutional autoencoder. The deep autoencoder model was trained to decode the original non-defect images from the defect images. The decoded images were then compared with the input image to identify the defect location. To overcome the small and imbalanced dataset in the early manufacturing stage, we applied appropriate image augmentation to improve the model training performance. The experimental results reveal that a simple unsupervised autoencoder model delivers promising performance, with a detection rate of up to 98% and a false pass rate below 1.7% for the test data, containing 3900 defect and non-defect images.

## 1. Introduction

A printed circuit board (PCB) mechanically supports the connection of electronic components via conductive tracks, pads, and soldering. PCB defects can cause malfunction and degrade the performance of the connected electronic components, which have a crucial impact on the performance of the entire system. Recently, in the mobile era, as the small mobile electronic product market has rapidly grown, more diverse and complicated PCB designs are required. This, in turn, produces PCB defect patterns that are difficult to detect by the human eye.

In general, PCB defect detection can be classified into two categories: direct inspection by a human operator and camera-based machine vision methods. Operator-based inspection allows operators to easily perform visual checks using simple instructions. However, operators can easily become fatigued by repetitive work and the detection results from each operator are not consistent. This is a fundamental limitation of human-based judgment and is the leading cause of defective products leaving the factory. To overcome these limitations, researchers have studied machine vision-based defect inspection, which consists of a camera, light source, and operation system. The main purpose of this approach is quality control using an automated optical inspection (AOI) system. The AOI system detects defects by acquiring high-quality images using an industrial camera such as Radiant vision camera [1], equipped with a charge-couples device (CCD) or complementary metal-oxide-semiconductor (CMOS) image sensor. In the past, CCD was more used due to the fixed pattern noise (FPN) of the CMOS sensor. However recently, CMOS sensors have been widely used because of their improved performance and lower price compared to CCD. There are three frequently used AOI approaches in PCB inspection: reference comparison, non-reference verification, and hybrid approaches [2]. The reference comparison method compares the images to be detected with the template images to obtain defect areas. It is intuitive and easy to understand but requires high alignment accuracy and is sensitive to the light environment of the photographing process. The non-reference comparison method checks whether the traces and layout of the circuit board to be tested are reasonable according to the design rules; however, this method can easily miss large defects and distortion characteristics. The hybrid comparison method considers both advantages, but it is difficult to implement and has a large amount of computational complexity.

Not only the methods from the abovementioned literature studies but also a wide range of machine vision and image processing algorithms are available for developers to utilize [3]. Ideally, an almost perfect AOI system can be developed if all the defect types are reported and studied in advance. However, one cannot guarantee that the system will encounter only preregistered defects. In a real production environment, new types of defects are always likely to be encountered and a typical machine vision-based detection system will not detect these correctly. In this case, the defect inspection system must be recalibrated using new sample data whenever the manufacturing conditions change [4]. This can be a major disadvantage of traditional machine vision-based inspection systems because process changes occur every year in recent manufacturing environments.

Recently, the advent of deep learning techniques has enabled developers to obtain more generalized computer and machine vision solutions. In particular, convolutional neural networks (CNNs) have yielded significant improvements in the image recognition and detection field [5]. A CNN can learn image features automatically and is advantageous in that it can operate without conjugating techniques for extracting features [6]. AlexNet, a competitor in ImageNet LSVRC-2012 and one of the most popular CNN structures, won with an error rate 10% lower than that of the computer vision model that won in the previous year [7]. In addition, the performances of CNNs appear to approach the levels of humans in recognition tasks [8]. Autoencoders [9,10] are another line of neural network structures that compress the input data into a low-dimensional representation and expand it to reproduce the original input data [11]. It is known that an autoencoder learns the structure of the image and reconstructs the original image from the corrupted input image. This motivated us to investigate the autoencoder as a PCB defect detection application. Herein, we propose a CNN-based autoencoder model that can effectively detect PCB defects by capturing images of the PCB with an industrial camera equipped with an image sensor such as a CMOS sensor without any prior knowledge of the defects or of the expert engineers’ normal/defect assessments.

## 2. Materials and Methods

### 2.1. Data

**Figure 1:**
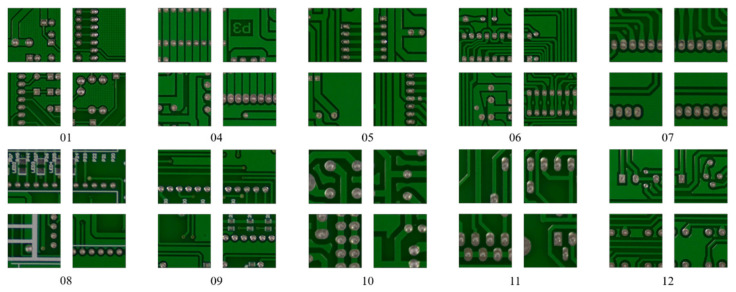
The 400×400 PCB images are cropped from Huang and Wei’s PCB dataset [12]. The number below each PCB image is the name of the reference PCB. The numbers below the cropped images are the name of the reference image files.

Huang and Wei created a dataset for PCB defects [12]. To verify our PCB defect detection method, we applied their open PCB defect dataset to our experiment. Ten reference PCBs were selected to create this dataset, each of which was captured by a 16-megapixel HD industrial camera equipped with a CMOS sensor. The resolution of the original image was 4608×3456 pixels and was adjusted according to the size of each PCB. The reference PCB images are presented in Figure 1.

**Figure 2:**
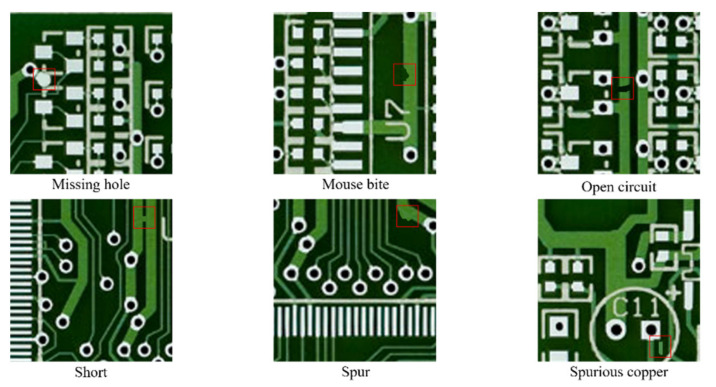
Examples of PCB defects.

After capturing the reference PCB images, we created artificial defects on the PCB images using Photoshop, a graphics editor published by Adobe Systems. There are six types of defects defined in this process: missing hole, mouse bite, open circuit, short, spur, and spurious copper. The images containing defects are labeled as defect classes, and each defect-labeled image has three to five defects of the same category in different places. The overall PCB dataset configuration is listed in Table 1. The dataset contains 693 PCB defect images, with 2953 defects that have been correctly labeled. The region including these defects was cropped to a fixed size of 400×400, and the entire cropped images consisted of 2953 images. Samples of the cropped defect images are shown in Figure 2.

### 2.2. Overall System Configuration

**Figure 3:**
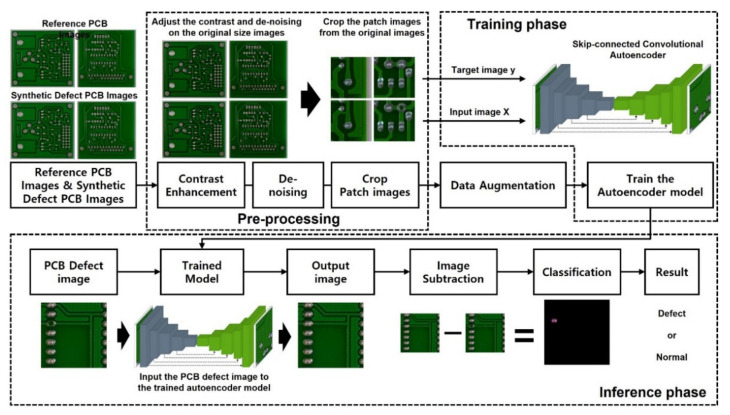
System overview of PCB defect detection (To enhance the quality of the data, we applied preprocessing to the PCB dataset. The quantity of the data for training is fulfilled by the data augmentation step, and all the data are inputted into our proposed autoencoder model. After the training, the trained model generates a non-defect image from the defect image, and image subtraction between these two images enables us to find the exact defect shape and location).

The pipeline of the entire PCB defect detection system proposed in this study is shown in Figure 3. In the preprocessing step, the original PCB dataset is processed by image contrast enhancement and noise rejection to improve the image quality. In the last step of the preprocessing, the PCB images are segmented into patch images of size 400×400 pixels, and these patch images including the defects are grouped separately for data augmentation. In the data augmentation block, defect patch data are augmented by random rotation, random flip, and random Gaussian to overcome the limitation resulting from a lack of data and an imbalance of data classes. Then, the augmented patch images, including the defects, are used to train the skip-connected convolutional autoencoder models [2] to predict the non-defect patch images of the input. Once the trained model predicts a high-quality PCB image from the defective PCB images, the defect detection map is generated by subtracting the infrared image from the defective input image. Critical defects can be highlighted by applying the appropriate threshold to the detection map images.

### 2.3. Preprocessing

Data preprocessing involves making a dataset suitable for training, and the quality of the training data determines the performance of the neural network models [13]. In this study, the PCB defect datasets were preprocessed for two main purposes. The first was to improve the quality of the images through clear contrast and noise filtering, and the second was to extract the defect area.

A clear contrast of the data is obtained by histogram equalization. Histogram equalization is a typical image enhancement method, and its operation is processed by remapping the grayscale levels of the image on the basis of the probability distribution of the input grayscale levels [14]. To remove the overall noise of the data, we apply a median filtering method. A median filter is a rank selection filter that has shown excellent ability to denoise salt and pepper noise [15]. The algorithm for the median filter is as follows:

Step 1. Select a two-dimensional window W of size 3 × 3. Assume that the pixel being processed is Cx,y.

Step 2. Compute Wmed, the median of the pixel values in window W.

Step 3. Replace Cx,y with Wmed.

Step 4. Repeat steps 1 to 3 until all the pixels in the entire image are processed.

High-resolution images are acquired by an industrial-grade camera, which requires high computational power and results in a long processing time in training the deep neural network model. Thus, preprocessing is designed to automatically obtain a specified patch image of size 400×400 from the original image, as well as to perform image contrasting and noise rejection.

### 2.4. Data Augmentation

Typically, training deep neural networks requires large-scale training data owing to the significantly large hyperparameters. However, the frequency at which defective products typically occur during the manufacturing process is bound to be small, and the types of defects can also change during mass production. This data imbalance issue can be a fundamental limitation of deep neural network-based defect inspection systems because mass production requires an appropriate inspection system before the production starts. Applying these unbalanced data to a deep neural network model can lead to several problems such as overfitting and performance degradation [16]. To avoid these problems, we applied data augmentation to supplement a small amount of defect data and improve the model performance. Considering the redundancy of the augmented images, geometric transformations and noise injection were applied. Geometric transformations are efficient methods for positional biases of the training data using variations in the shape, orientation, or location of the part features. Random rotation and random flip are applied to overcome the positional biases of the PCB data. Noise injections are methods that involve adding or multiplying a matrix of random values from a noise distribution, and a random Gaussian noise function is applied to generate this noise to help neural network models learn more robust features.

### 2.5. Skip-Connected Convolutional Autoencoder

**Figure 4:**
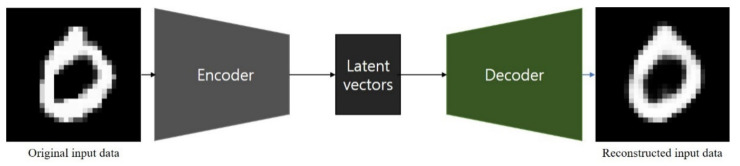
Working process of an autoencoder: transforming input data to compressed latent vectors and then decoding it as the data.

An autoencoder is a network that aims to encode an input to a low-dimensional latent space and then decode it [17]. It is an unsupervised learning algorithm that allows the extraction of generally useful features from unlabeled data [18]. As shown in Figure 4, an autoencoder consists of two parts: an encoder, which transforms the input data into low-dimensional latent vectors, and a decoder, which expands the latent vectors to reproduce the original input data. They are commonly used for data compression [19,20], denoising [21,22,23], and anomaly detection [24,25,26,27].

Because typical autoencoders, such as fully connected autoencoders, ignore the two-dimensional (2D) image structure [28], an autoencoder consisting of convolutional layers (Conv) is used for dealing with 2D image data. This is called a convolutional autoencoder. Conv are core components of CNNs, which have been commonly applied to analyze visual imagery, with each layer of parameters being composed of learnable filters. When the input data pass through the Conv, the resistance between the filter and the input data is calculated through a voltage operation at the width and height of the input volume, and a feature map is generated in two dimensions through the activation function such as the rectified linear unit (ReLU) and sigmoid functions [29]. It provides model flexibility.

(1) $$a_{out}=max\left(a_{in}^{n\times n}u\left(n,n\right)\right)$$

The main purpose of the pooling layer is to maintain spatial permanence while reducing the resolution of the feature map, which allows efficient learning by reducing the amount of computation by decreasing the size of the data and feature maps [29]. Generally, max pooling is used frequently and the window function $u\left(x,y\right)$ of (1) is applied to the input $a_{in}^{n\times n}$, replacing each neighborhood of the input with the maximum value $a_{out}$, reducing the size of the input.

**Figure 5:**
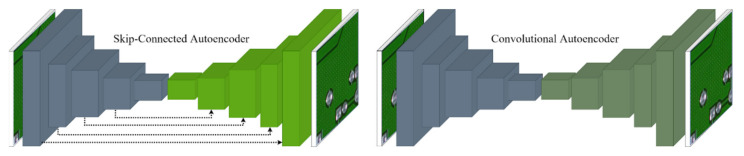
Architectures of the skip-connected convolutional autoencoder and convolutional autoencoder. The arrows indicate the skip connections.

According to [30], when deeper networks can start converging, a degradation problem is encountered. This problem saturates the accuracy of the network as the network depth increases. When the autoencoder encounters this problem, it is difficult to learn the details from the data. To address this, we added skip connections between the two encoder and decoder layers, as shown in Figure 5. The skip connections between the corresponding encoder and decoder layers allows to converge to a better optimum in pixel-wise prediction problems [9]. Let the outputs from the encoder layer and the corresponding decoder layer be $X_{1}$ and $X_{2}$, respectively. The input to the next decoder layer is calculated as follows:

(2) $$F\left(X_{1},X_{2}\right)=X_{1}⊕X_{2}$$

Through skip connections, each feature map of the corresponding encoder and decoder are summed element-wise, which helps the network to recover the image well. The autoencoder used in this study was trained to reproduce the non-defect image data from the defect image data. Table 2 shows the overall architecture of the proposed autoencoder.

As shown in Table 2, the layers above and below the table center line are the encoder and decoder parts, respectively. The input data of size 400×400×3 are encoded into latent vectors through the encoder part, and the decoder generates the output data, which has the same size as that of the input data from the latent vectors. Through this encoder, the decoder process reproduces the defect image as an image without the defect and the generated image is used for image subtraction for defect detection. Each convolutional layer includes the ReLU activation function and batch normalization. The ReLU activation function allows models learn fast, makes models learn qualitatively sensible features from data. [31] Each skip connection complements the data loss due to the data compression in the encoder part by combining the encoder Conv output and the UpSampling output.

The proposed skip-connected convolutional autoencoder has 26.8 BFLOPs to process a 400×400 image. This is 2.24 times less compared to the famous real-time object detection model called yolo v4 to process a 416×416 image. Yolo v4 runs at 55 FPS when using an NVIDIA RTX 2070 as a computational unit, from which we can see that the skip-connected convolutional autoencoder can run inference in real time faster than yolo v4.

### 2.6. Performance Evaluation

In our approach, our model generates a non-defect output image and subtracts it from the input image. This image subtraction method is a process whereby the digital numeric value of the whole image is subtracted from that of another image [32]. Through this method, we can detect changes between two images and this detection of changes can be used to recognize defects. Therefore, the better quality of output images indicates the performance improvement of our model, and this performance indicator measures how similar it is to the target image. The mean square error (MSE) and peak signal-to-noise ratio (PSNR), which calculates similarity with absolute difference in pixel values, can be calculated with high similarity even for the blurred image, we applied the structural similarity index measurement (SSIM). SSIM measures the degradation of the structural information in one image compared with that of another image. Specifically, SSIM is calculated first by comparing the luminance, contrast, and structural similarities between two images. The standard SSIM is calculated as follows:

(3) $$SSIM\left(x,y\right)=\frac{\left(2\mu_{x}\mu_{y}+c_{1}\right)\left(2\sigma_{xy}+c_{2}\right)}{\left(\mu_{x}^{2}+\mu_{y}^{2}+c_{1}\right)\left(\sigma_{x}^{2}+\sigma_{y}^{2}+c_{2}\right)}$$

where μ and σ denote the local mean and local variance, respectively; $\sigma_{xy}$ is the local covariance; and $c_{1}$, $c_{2}$ are constants to prevent division by zero.

Accuracy is the most basic indicator used to evaluate detection and classification models. However, because general accuracy does not consider class imbalances in the data, accurate performance evaluations can be difficult. Therefore, different metrics should be considered when evaluating the predictive accuracy of each class. In this study, which deals with unbalanced data, widely employed indicators were utilized for the model evaluation [33].

The performance evaluation indicators used in this study were the accuracy, true positive rate (*TPR*), true negative rate (*TNR*), precision, F1 score, and balanced classification rate (BCR), which all take percentage values between 0 and 1, where a value closer to 1 represents a better performance. Each performance indicator utilizes the components of the confusion matrix shown in Table 3. NG represents all defects, and OK represents a non-defective case. The defect class was considered to be positive in this study. The accuracy, calculated using Equation (4), represents the ratio of the total data predicted accurately by the model and is the most fundamental metric for evaluating the model.

The *TPR* and *TNR*, calculated using Equations (5) and (6), respectively, represent the rates at which the model correctly identifies the actual data as defective and normal, respectively. The precision is calculated using Equation (7) and represents the ratio of actual defects to defects predicted from the data. Using the *TPR*, *TNR*, and precision, we can determine how accurately the normal and defective cases are predicted.

(4) $$Accuracy=\frac{TP+TN}{TP+FN+TN+FP}$$

(5) $$TPR=\frac{TP}{TP+FN}$$

(6) $$TNR=\frac{TN}{TN+FP}$$

(7) $$Precision=\frac{TP}{TP+FP}$$

(8) $$F1\;score=\frac{2}{\frac{1}{Precision}+\frac{1}{Recall}}=\frac{2\times \left(Precision\times Recall\right)}{\left(Precision+Recall\right)}$$

(9) $$BCR=\frac{1}{2}\times \frac{TP}{TP+FN}+\frac{TN}{TN+FP}$$

The *F1 score* and *BCR* are indicators that summarize the classification performance, and they are given by Equations (8) and (9), respectively. The F1 score is the harmonic mean of the precision and *TPR*, whereas the *BCR* is the geometric mean of the *TPR* and *TNR*. The models used for the performance evaluation were the proposed skip connected convolutional autoencoder and a convolutional autoencoder. The values obtained for each model were calculated using the softmax values of each class. Then, the highest value was determined as the final result, and all the results were aggregated to calculate the performance metrics on the basis of the confusion matrix in Table 3.

## 3. Experiment Results

**Figure 6:**
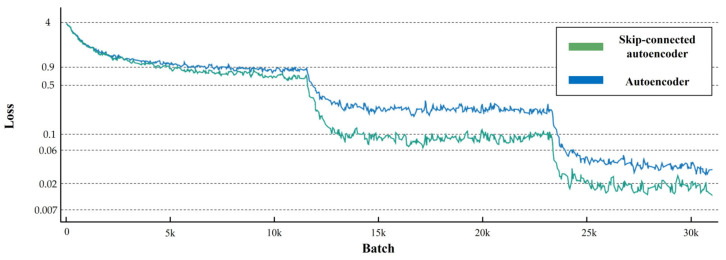
Training losses of the two models. The green line graph is the training loss of the skip-connected convolutional autoencoder, whereas the blue one indicates that of the convolutional autoencoder. Both models show decreased training loss according to the learning rate.

To train the models, we applied data augmentation to the dataset. The augmented dataset consisted of 98,730 patch images, and each patch image was paired with a reference normal patch image. We followed the training hyperparameter settings in [34], as listed in Table 4. The initial learning rate was 0.1, which was divided by 5 at 60, 120, and 160 epochs; the weight decay was 5 × 10^−4^; and the Nesterov momentum was 0.9. The total number of training epochs was 300 with a batch size of 128. Figure 6 shows the training losses of the two networks. As shown in the figure, the training losses decrease rapidly according to the learning rate schedule and the training loss of the skip-connected convolutional autoencoder is slightly lower than that of the autoencoder. As we mentioned above, the lower training loss in the skip-connected autoencoder is because skip connections solve the degradation problem so that it can converge to a better optimum.

**Figure 7:**
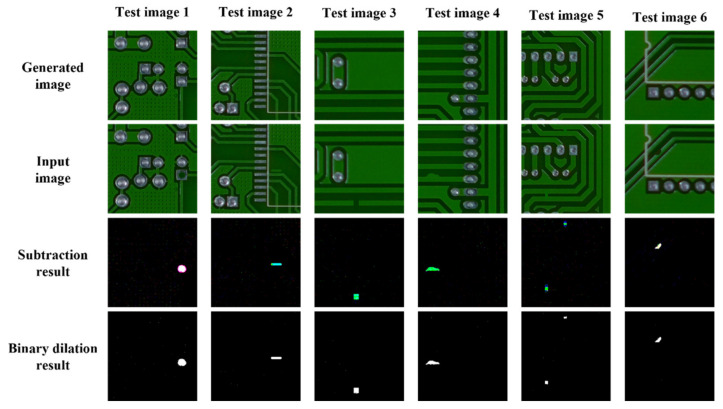
Results from the six test images. The subtraction result is the difference between the generated image and the input image. The binary dilation result is an image whose noise is removed by using the dilation technique.

To measure the performance of both training models at 300 epochs, we used a test dataset that was not used during the training to test the models. As shown in Table 5, the test dataset contains 3900 patch images, which consist of 2000 defect images and 1900 normal images. The trained models used the test data to generate artificial non-defect images and performed image subtraction to check the difference between the generated images and the input images. Figure 7 shows a sample result of the model test with six test images. The difference between the two pairs of images is an indicator of defects or a normal surface depending on the amount of difference; therefore, if the output images are almost the same as the input images, it will have an adverse effect on the classification performance of the model. To increase the quality of the images for defect detection, we applied image thresholding to filter out extra noise and highlight the defect area.

**Figure 8:**
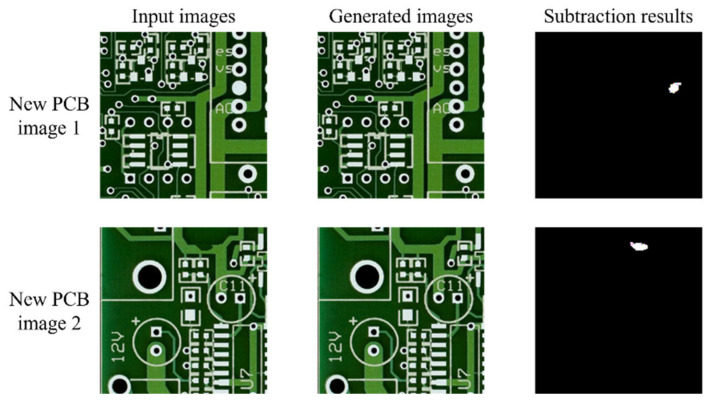
Results from the new PCB images. The defects of input images were artificially drawn.

The performance evaluation results for both models using the test dataset with 3900 patch images are shown in Table 6. To compare the defect classification performance, we calculated the accuracy, TPR, TNR, precision, F1 score, and BCR. SSIM was calculated to compare which model has a better non-defect image regeneration performance. As shown in Table 6, the accuracy of the skip-connected convolutional autoencoder is approximately 3% higher than that of the convolutional autoencoder. In addition, the SSIM of the former autoencoder is higher than that of the latter autoencoder. The skip-connected convolutional autoencoder works well with various defect types. Additionally, we tested the skip-connected convolutional autoencoder using new PCB images. As shown in Figure 8, the skip-connected convolutional autoencoder works well for other PCB images.

## 4. Conclusions

In this paper, we proposed a PCB defect inspection system based on a skip-connected convolutional autoencoder. The PCB datasets were preprocessed to reduce the image size by removing the unused area and to improve the image quality using well-known image enhancement algorithms. In addition, datasets were augmented to mitigate the issue of an imbalanced training dataset. The pretrained and augmented datasets can successfully train the autoencoder model to predict the non-defect images from the potentially defective PCB sample images. Finally, we evaluated the model performance using the SSIM, which measures the similarity between the model output and the target. According to the SSIM evaluation result, the addition of the skip connection to the autoencoder increases the similarity and the prediction performance of the model as well.

Lastly, we used image subtraction to obtain the difference between the non-defect output image and the input image. The difference between the model input and the output image is crucial information for detecting PCB defects and their exact locations.

Although we achieved highly accurate test results based on synthetic defect data, there are few issues that need to be overcome in the future. First, because we used artificially generated PCB defect images, it is necessary to validate our model on real PCB defect data to confirm the performance of our model in an actual measurement environment. Second, because the defect patterns in the datasets were synthesized on the basis of a few well-known defect patterns, our method might show a low detection rate for untrained defect datasets.

## Figures and Tables

**Table 1:** Overall PCB dataset configuration. The adjusted size is the size adjusted from the original 4608×3456 resolution to the size of each reference PCB. The number in parentheses of the total refers to the number of artificially generated defects.

Reference PCB Name	Adjusted Size	Defect Type					
		Missing Hole	Mouse Bite	Open Circuit	Short	Spur	Spurious Copper
1	3034 × 1586	20	20	20	20	20	20
4	3056 × 2464	20	20	20	20	20	20
5	2544 × 2156	10	10	10	10	10	10
6	2868 × 2316	10	10	10	10	10	10
7	2904 × 1921	10	10	10	10	10	10
8	2759 × 2154	10	10	10	10	10	10
9	2775 × 2159	10	10	10	10	10	10
10	2240 × 2016	10	10	10	10	10	10
11	2282 × 2248	10	10	10	10	10	10
12	2529 × 2530	10	10	10	10	10	10
Total image (total number of defects)		115 (497)	115 (492)	116 (482)	116 (491)	115 (488)	116 (503)
		693 (2593)					

**Table 2:** Overall architecture of the skip-connected convolutional autoencoder. In the kernel column, the first number denotes the number of channels produces by the convolution, and the tuple of two number denotes the filter size.

Layer	Kernel	Output
Input	–	(400, 400, 3)
Conv1	64, (5,5)	(400, 400, 64)
MaxPooling1	(2,2)	(200, 200, 64)
Conv2	64, (5,5)	(200, 200, 64)
MaxPooling2	(2,2)	(100, 100, 64)
Conv3	128, (3,3)	(100, 100, 128)
MaxPooling3	(2,2)	(50, 50, 64)
Conv4	128, (3,3)	(50, 50, 64)
MaxPooling4	(2,2)	(25, 25, 128)
Conv5	128, (3,3)	(25, 25, 128)
UpSampling1	(2,2)	(50, 50, 128)
Conv6	128, (3,3)	(50, 50, 128)
UpSampling2	(2,2)	(100, 100, 128)
SkipConnection1	–	UpSampling2 + Conv3
Conv7	64, (5,5)	(100, 100, 64)
UpSampling3	(2,2)	(200, 200, 64)
SkipConnection2	–	UpSampling3 + Conv2
Conv8	64, (5,5)	(200, 200, 64)
UpSampling4	(2,2)	(400, 400, 64)
SkipConnection3	–	UpSampling4 + Conv1
Conv9	3, (3,3)	(400, 400, 3)

**Table 3:** Confusion matrix of the defect classification.

		Actual Class	
		NG	OK
Predicted Class	NG	True Positive (*TP*)	False Positive (*FP*)
	OK	False Negative (*FN*)	True Negative (*TN*)

**Table 4:** Training hyperparameter settings of the autoencoder and skip-connected autoencoder.

Hyperparameter	Value				
Total number of epochs	300				
Batch size	128				
Optimizer	Weight decay: 5 × 10^−4^				
	Momentum: 0.9				
Learning rate (lr)	Epoch	60	120	160	300
	Lr	0.1	0.02	0.004	0.0008

**Table 5:** The dataset configuration. The training data are for the autoencoders, whereas the test data are for the defect existence classification test.

Dataset Configuration		
Total number of training data	98,730 pairs of defect and non-defect images	
Total number of test data	3900	
	1900 normal images	2000 defect images

**Table 6:** Comparison between the convolutional autoencoder and the skip-connected convolutional autoencoder in terms of defect classification performance.

Models	Accuracy	TPR	TNR	Precision	F1	BCR	SSIM
Convolutional autoencoder	0.9508	0.9131	0.9865	0.9847	0.9475	0.9491	0.9510
Skip-connected convolutional autoencoder	0.9808	0.9773	0.9840	0.9830	0.9801	0.9806	0.9749

## Data Availability

The data presented in this study are included within the article.

## Abbreviations

The following abbreviations are used in this manuscript:

PCB	Printed Circuit Boards
AOI	Automated Optical Inspection
CCD	Charge Coupled Device
CMOS	Complementary Metal Oxide Semiconductor
CNN	Convolutional Neural Network
Conv	Convolutional Layers
ReLU	Rectified Linear Unit
BFLOPs	Billion Floating-point Operations
FPS	Frames Per Second
MSE	Mean Square Error
PSNR	Peak Signal-to-Noise Ratio
SSIM	Structural Similarity Index Measurement
TPR	True Positive Rate
TNR	True Negative Rate
BCR	Balanced Classification Rate
